# Discrete Element Modeling of Concrete Under Dynamic Tensile Loading

**DOI:** 10.3390/ma18143347

**Published:** 2025-07-17

**Authors:** Ahmad Omar, Laurent Daudeville

**Affiliations:** 1 Faculty of Engineering, Lebanese University, Beirut P.O. Box 6573/14, Lebanon; ahmad.omar.edu@gmail.com; 2 University Grenoble Alpes, CNRS, Grenoble INP, 3SR, 38041 Grenoble, France

**Keywords:** discrete element model, concrete, spalling test, strain rate effect, dynamic tensile behavior

## Abstract

Concrete is a fundamental material in structural engineering, widely used in critical infrastructure such as bridges, nuclear power plants, and dams. These structures may be subjected to extreme dynamic loads resulting from natural disasters, industrial accidents, or missile impacts. Therefore, a comprehensive understanding of concrete behavior under high strain rates is essential for safe and resilient design. Experimental investigations, particularly spalling tests, have highlighted the strain-rate sensitivity of concrete in dynamic tensile loading conditions. This study presents a macroscopic 3D discrete element model specifically developed to simulate the dynamic response of concrete subjected to extreme loading. Unlike conventional continuum-based models, the proposed discrete element framework is particularly suited to capturing damage and fracture mechanisms in cohesive materials. A key innovation lies in incorporating a physically grounded strain-rate dependency directly into the local cohesive laws that govern inter-element interactions. The originality of this work is further underlined by the validation of the discrete element model under dynamic tensile loading through the simulation of spalling tests on normalstrength concrete at strain rates representative of severe impact scenarios (30–115 s^−1^). After calibrating the model under quasi-static loading, the simulations accurately reproduce key experimental outcomes, including rear-face velocity profiles and failure characteristics. Combined with prior validations under high confining pressure, this study reinforces the capability of the discrete element method for modeling concrete subjected to extreme dynamic loading, offering a robust tool for predictive structural assessment and design.

## 1. Introduction

Concrete is extensively used in civil engineering, particularly in the construction of large-scale infrastructure such as dams and nuclear power plants. The design of these critical structures must account for extreme natural or human-induced hazards that may lead to catastrophic failure. Among these are dynamic events such as explosions, missile impacts, and aircraft crashes. Current design methodologies for concrete structures under such conditions often rely on empirical formulations derived from costly full-scale experiments [1,2,3,4]. However, these formulations typically have limited applicability beyond the specific loading regimes for which they were calibrated. Consequently, the development of advanced numerical tools is essential for achieving accurate and predictive modeling of concrete behavior under extreme dynamic scenarios.

The finite element method (FEM) is widely used to simulate complex non-linear problems in structural engineering. However, under extreme dynamic loading, structures can undergo significant fragmentation and cracking, phenomena that FEM, as a continuum-based method, may struggle to represent accurately. Although various FEM-based approaches have been proposed for high-impact simulations, they often rely on erosion criteria with limited physical justification and require case-specific calibration [5,6].

As an alternative, the discrete element method (DEM) offers a natural framework for modeling discontinuities, making it particularly suited to simulate damage evolution and fragmentation in cohesive materials. Since the foundational work of Cundall and Strack [7], DEM has been successfully applied to a range of materials including granular media, rocks, ceramics, and concrete [8,9,10,11,12]. However, existing DEM models typically do not account for both rate-dependent brittle failure in tension and ductile compaction in compression—two key features of concrete subjected to severe dynamic loading.

This study presents a robust and versatile DEM model capable of reliably reproducing the behavior of concrete under extreme loading. After validating the DEM model for high triaxial compresion stress states by simulations of missile penetration tests performed on concrete slabs [13], the focus is placed on the tensile response of concrete under strain rates representative of hard impact scenarios. The model, originally developed by Daudeville et al. [13,14,15,16,17] has been implemented within Europlexus [18], a finite element software jointly developed by the French Alternative Energies and Atomic Energy Commission (CEA) and the European Commission’s Joint Research Centre. Europlexus is specifically designed for fluid–structure interaction analyses under transient dynamic conditions.

The strain-rate dependency of tensile concrete strength has been extensively documented. Malvar et al. [19] introduced the Dynamic Increase Factor (DIF), which quantifies the ratio of dynamic to static strength. They modeled the DIF as a bilinear function of the strain rate, identifying distinct regimes for moderate and high rates, with a transition occurring around 1 $s^{-1}$. Other studies have shown that fracture energy increases with strain rate [20] and that dynamic softening behavior tends toward increased brittleness [21]. In contrast, the rate effects observed in compression are significantly less pronounced [22]. Cusatis [23] demonstrated that in unconfined compression tests at strain rates exceeding $10^{-1}$ $s^{-1}$, inertial effects contribute substantially to apparent strength gains. Accurately modeling these strain-rate effects is essential for simulating dynamic fracture processes in concrete.

**Figure 1:**
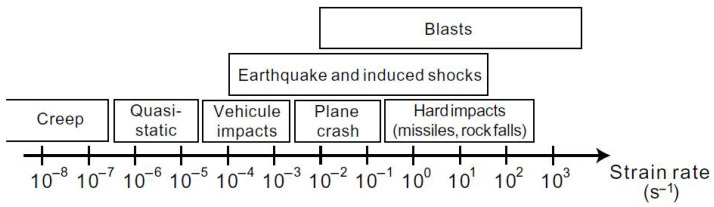
Typical strain rates in concrete for various types of loading [22].

The originality of this work lies in the identification and validation of the model parameters for normal-strength concrete subjected to dynamic tensile loading, with strain rates ranging from several tens to several hundreds of $s^{-1}$. These conditions are representative of hard impacts (Figure 1). In particular, the model is validated through simulations of spalling tests performed on a reference normal strength concrete by Erzar [24,25]. After calibrating the model under quasi-static conditions, the introduction of a strain-rate law enables the simulation of dynamic fracture processes with improved accuracy.

The paper is organized as follows: Section 2 reviews the experimental procedures used to characterize the tensile behavior of concrete at high strain rates, focusing on modified split Hopkinson pressure bar (SHPB) tests and the results of experiments performed by Erzar [24,25]. Section 3 describes the DEM model developed for concrete and its key constitutive features. Section 4 details the parameter identification process using quasi-static laboratory tests performed on the reference normal strength concrete and dynamic test results from literature. Section 5 presents and discusses the simulation of the spalling tests. Finally, conclusions are drawn in Section 6.

## 2. Experimental Analysis of the Dynamical Tensile Behavior of Concrete

### 2.1. Testing Devices

Two primary categories of testing devices are employed to characterize the dynamic tensile behavior of concrete [26,27]. High-rate servo-hydraulic machines enable direct tensile testing of concrete at strain rates up to approximately 1 $s^{-1}$. These systems are well-suited for testing concrete in the moderate strain-rate regime. However, at higher strain rates, the assumption of quasi-static equilibrium within the specimen becomes invalid, complicating both the test execution and the interpretation of results. Additionally, the limited acceleration capacity of hydraulic jacks makes it challenging to impose a stable crosshead velocity and maintain a consistent loading rate prior to failure. Another issue is the limited acceleration of the jack’s hydraulic presses that makes difficult to impose a stabilized velocity of the moving crosshead and a constant loading rate in the sample before failure. To overcome these limitations, modified split-Hopkinson pressure bar (SHPB) setups are commonly used to investigate the tensile behavior of concrete at high strain rates, typically between $10^{1}$ to $2.10^{2}$ $s^{-1}$ [20,28,29,30,31,32,33,34,35,36]. Plate impact experiments are generally employed to characterize material behavior at higher strain rates [37].

**Figure 2:**
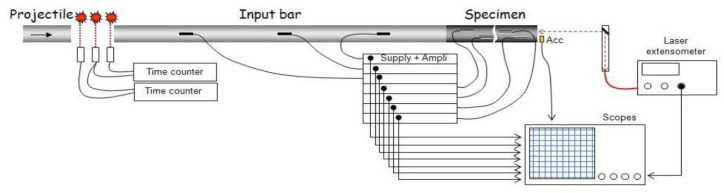
Modified SHPB set-up and instrumentation [24].

Several SHPB configurations have been developed [26,28]; this study focuses on spalling tests using a single Hopkinson bar in contact with a cylindrical concrete specimen of identical diameter [38,39] (Figure 2). The projectile and the bar are made from aluminum alloy, selected for its impedance compatibility with concrete, which minimizes signal reflections at the specimen–bar interface. In this method, the specimen is placed with one face in contact with the bar and the opposite face left free. A compressive pulse is generated on the bar side and propagates through the specimen. Upon reaching the free end, the compressive wave reflects as a tensile pulse. If the amplitude of the reflected tensile wave exceeds that of the incident compressive wave, dynamic tensile stresses develop in the core of the specimen. When these stresses exceed the material’s tensile strength, spalling occurs and the specimen fractures.

Klepaczko and Brara [38] proposed two methods for determining the dynamic tensile strength. In the first method, the measures of the incident and reflected signals on the Hopkinson bar allow reconstructing the transmitted compressive pulse and infer the internal tensile stress distribution as a function of time. The tensile strength is then obtained at the fracture location. The second method consists in measuring the residual velocity of fragments and deducing the spall strength from a one-dimensional elastic-wave analysis.

**Figure 3:**
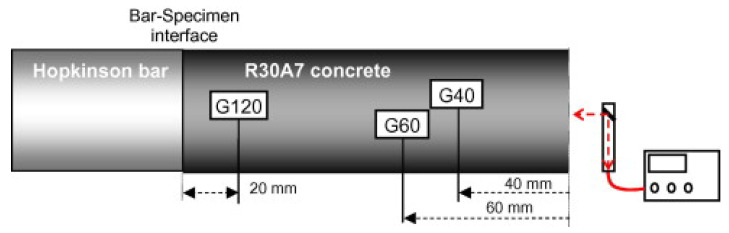
Strain gauges and interferometers used in spalling tests [24].

However, Erzar and Forquin [39] noted that both approaches can introduce inaccuracies. The first method suffers from difficulty in localizing the fracture due to damage spreading over several centimeters, while the second method faces challenges in precisely measuring particle velocities on both sides of the fracture plane. To address these limitations, Schuler et al. [20] and Erzar et al. [25,39] adopted an alternative method that uses the velocity signal recorded at the free end of the specimen (Figure 2 and Figure 3). They employed the formula proposed by Novikov et al. [40] to calculate the spall strength (1).

(1) $$\sigma_{F}=\frac{1}{2}\;\rho V_{0}\;\Delta V_{pb}\;$$

**Figure 4:**
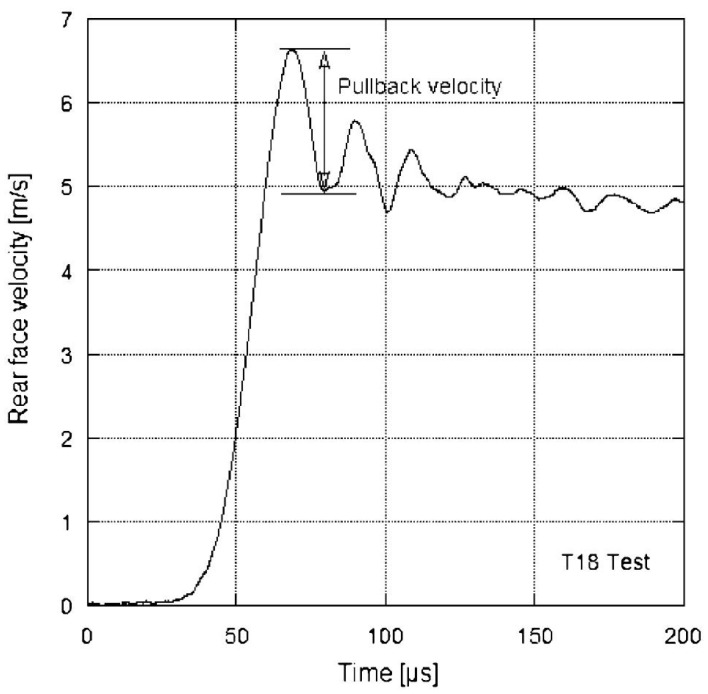
Particle velocity at the rear face of the specimen versus time deduced from the laser extensometer measurement. Test performed on MB50 micro-concrete by Erzar [24].

$\Delta V_{pb}$ is the pullback velocity, defined as the difference between the maximum rear-face velocity and the velocity at the first rebound (Figure 4). Subsequently, ρ is the concrete density and $V_{0}$ is the 1D wave speed, calculated from the time difference between the peak signal on a strain gauge and the corresponding peak at the free end of the specimen (Figure 4). Equation (1) is derived from the momentum equation for elastic stress wave propagation in a one-dimensional bar. It is valid if the material behaves elastically, i.e., the compressive wave does not provoke any dissipative phenomena and the tensile behavior is elastic and brittle.

The test setup is comprehensively instrumented. Photodiode beams measure the projectile’s impact velocity, and strain gauges placed along the Hopkinson bar record incident and reflected waves (Figure 2). Additional strain gauges are affixed directly to the specimen. All measurements are synchronized via high-frequency acquisition systems. Two laser-based extensometers using the Doppler effect are employed to capture rear-face velocities and particle velocities near the specimen–bar interface (Figure 2 and Figure 3). Using this setup, Erzar [24,25] conducted spalling tests on normal strength concrete specimens at strain rates reaching 150 $s^{-1}$.

### 2.2. Spalling Tests Performed on Normal Strength Concrete

The reference normal strength concrete used in this study, designated R30A7, has been extensively investigated at the 3SR laboratory of University Grenoble Alpes under both quasi-static triaxial loading [41,42] and dynamic conditions [25,43]. The composition and mechanical properties of R30A7 are provided in Table 1.

**Figure 5:**
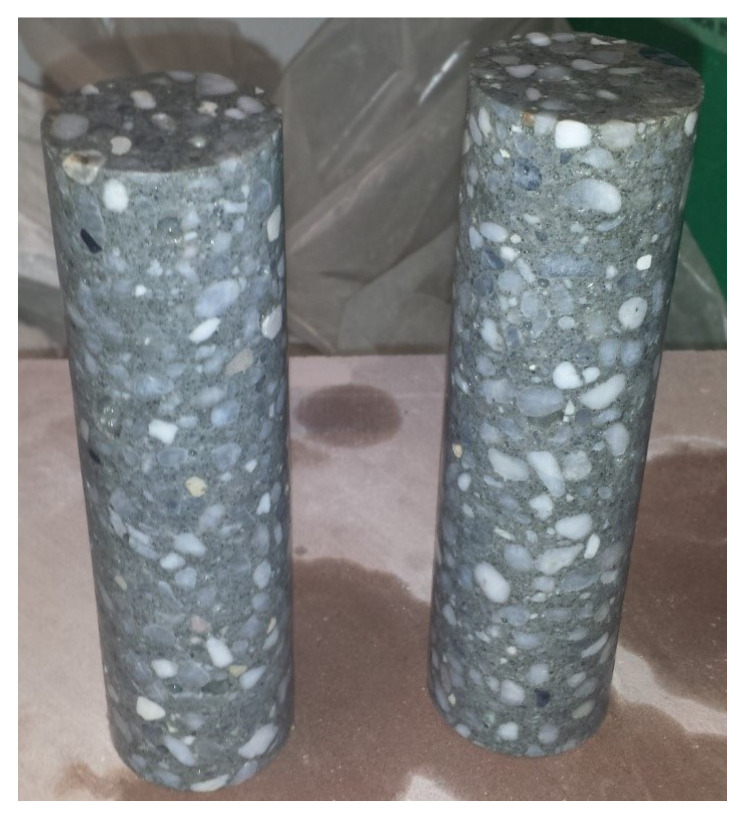
R30A7 concrete specimens.

The four spalling tests analyzed in this study were performed by Erzar [24,25] on R30A7 concrete cylindrical specimens, each measuring 45.7 mm in diameter and 140 mm in length. To minimize desiccation-induced damage and ensure material consistency, the specimens were tested in a wet condition. They were drilled from large concrete blocks that had been cast and submerged in lime-saturated water to prevent portlandite dissolution (Figure 5).

At the end of the preparation process, the degree of saturation was estimated at approximately 42%, based on the comparison between the mass of the wet specimen and that of a fully dried one.

The compressive stress–strain response and compressive strength at 28 days (34 MPa) were obtained from uniaxial compression tests, the tensile strength (3 MPa) was determined using the Brazilian splitting test [41].

During the spalling tests, the incident and reflected stress waves recorded in the Hopkinson bar were used to compute the compressive loading pulse transmitted to the specimen. Simultaneously, the velocity of the specimen’s rear face was measured using a laser extensometer (Figure 3). By combining these measurements, the strain rate at failure was determined for each of the four tests (labeled A to D), with values listed in Table 2.

**Figure 6:**
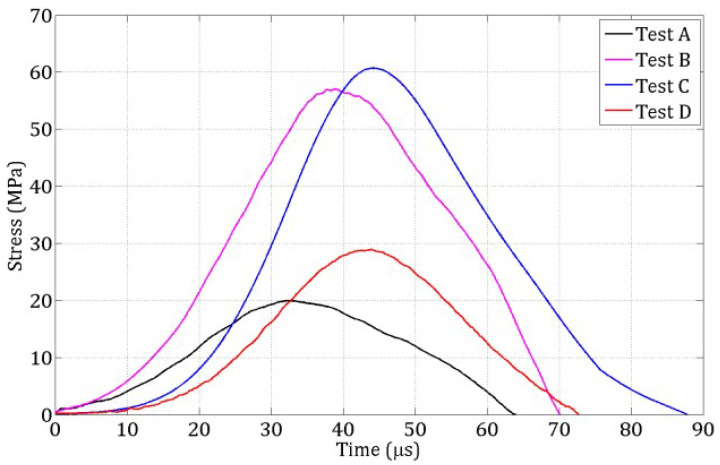
Stress pulses at the bar–specimen interface for the four spalling tests carried out on wet R30A7 concrete.

**Figure 7:**
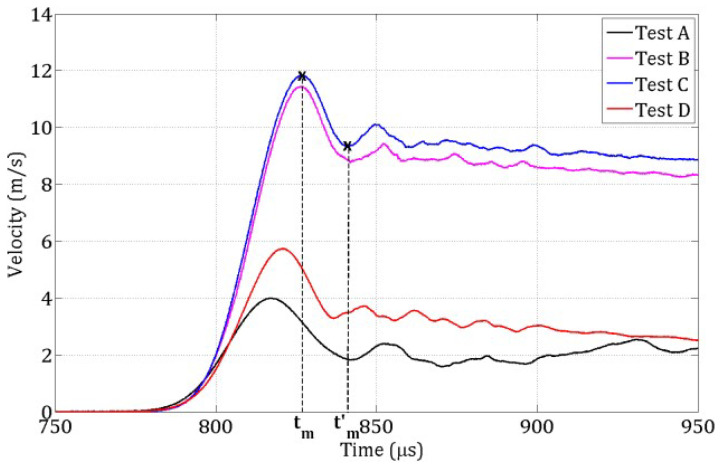
Rear face velocities for the four spalling tests carried out on wet R30A7 concrete.

The compressive stress pulses from these four tests are shown in Figure 6 and serve as input loading profiles for the numerical simulations. Figure 7 displays the evolution of the rear-face velocity, which is a key metric for validating the 3D DEM model. Specifically, the model’s accuracy in reproducing damage mechanisms is evaluated by its ability to predict the rear-face velocity profile, particularly: the maximum velocity at time $t_{m}$ and the rebound velocity at time $t_{m}^{\prime }$ which together define the pullback velocity, directly linked to the tensile failure stress.

## 3. DEM Model for Concrete

The DEM model used in this study is described in detail by Antoniou et al. [13]. This model includes ductile closure porosity under triaxial compression at high mean stress, brittle failure under tension and shear as well as brittle-ductile transition at intermediate mean stresses. The model accounts for the strain-rate-dependent dynamic tensile behavior. This article focuses on the constitutive behavior under tensile loading and its validation at high rates of loading. The model is implemented within the Europlexus software [18], an industrial finite element code specifically designed for transient dynamic simulations involving fluid–structure interaction. It employs an explicit central difference time integration algorithm, making it particularly well suited for modeling high-speed phenomena such as explosions and impacts.

Concrete is modeled as a homogeneous, isotropic material at the macroscopic scale. The DEM representation uses rigid spherical discrete elements (DE) where mass is concentrated. This choice minimizes the computational cost and allows easily handling interactions between DE. Each DE has six degrees of freedom, three translational and three rotational. These elements do not represent the actual mesoscale components of concrete (e.g., aggregates), but rather function at a higher-order scale to replicate the macroscopic behavior in both the linear and non-linear regimes.

Spring-like cohesive interactions are introduced between elements, which may or may not be in physical contact. The isotropic feature of the behavior requires an uniform distribution of the orientations of interactions between DE in all the directions of the space.

### 3.1. Discrete Element Generation and Packing Strategy

**Figure 8:**
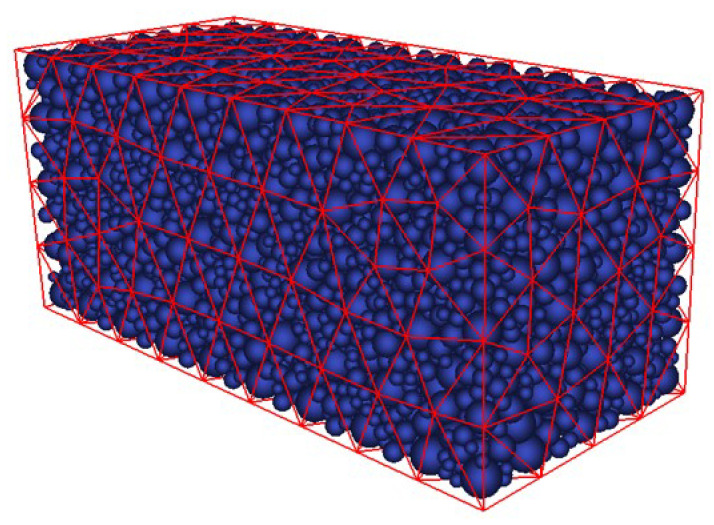
Tetrahedral FE mesh in red filled by spherical elements in blue.

To avoid artificial cleavage planes resulting from regular arrangements, the DEM assemblies incorporate spheres of varying sizes. Moreover, high packing density is critical to minimizing non-physical porosity. The packing of spheres is generated using a geometric algorithm developed by Jerier et al. [44], which fills a tetrahedral finite element (FE) mesh with non-overlapping spherical elements and then densifies the structure by inserting additional spheres into the voids (Figure 8). This algorithm yields dense, isotropic, and polydisperse assemblies with controlled size distributions at relatively low computational cost, while also allowing the creation of complex sample geometries.

### 3.2. Cohesive and Contact Interactions Between Elements

Once the assembly is created, two types of interactions are defined between DE: cohesive interactions and contact interactions activated when two elements physically touch.

**Figure 9:**
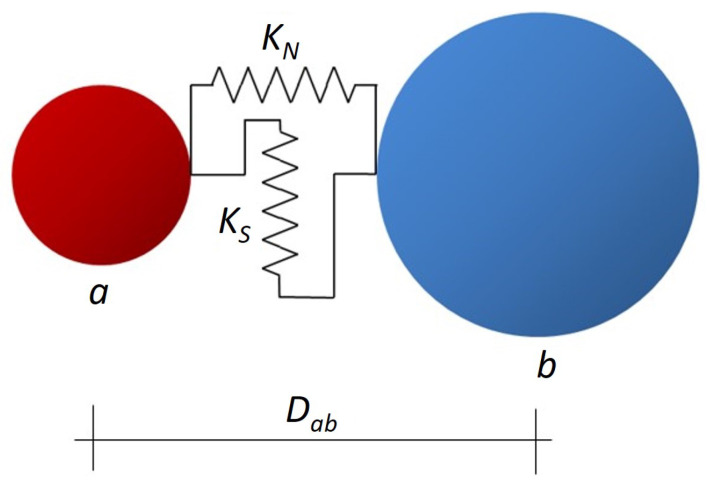
Cohesive interaction between DE *a* and *b*.

A cohesive link is established between two elements *a* and *b* of radii $R_{a}$ and $R_{b}$, respectively (Figure 9), if their separation distance $D_{ab}$ satisfies (2):

(2) $$\lambda \left(R_{a}+R_{b}\right)>D_{ab}$$

where λ≥1 is the interaction range. The value of λ is chosen such that each element has an average of 12 cohesive interactions. This empirical choice ensures the isotropy of the DE assembly.

The cohesive interactions are created initially to represent the cohesive feature of concrete. If a cohesive bond breaks and the DE come into contact, a contact interaction is established between the DE (λ=1). These contact interactions do not transmit tensile forces.

### 3.3. Elastic Behavior and Rolling Resistance

The elastic response of cohesive interactions is characterized by normal stiffness $K_{N}$ and tangential stiffness $K_{S}$. These are related to the macroscopic Young’s modulus *E* and Poisson’s ratio ν using the micro–macro relations (3) and (4) [16]:

(3) $$K_{N}=\frac{E.S_{int}}{D_{init}}\cdot \frac{1+\alpha }{\beta (1+\nu )+\gamma (1-\alpha \nu )}$$

(4) $$K_{S}=K_{N}\frac{1-\alpha \nu }{1+\nu }$$

where $D_{init}$ is the initial distance between the DE centroids and $S_{int}$ = π. Min($R_{a}^{2}$, $R_{b}^{2}$) denotes the interaction surface and α,β and γ are dimensionless parameters dependent on the packing.

**Figure 10:**
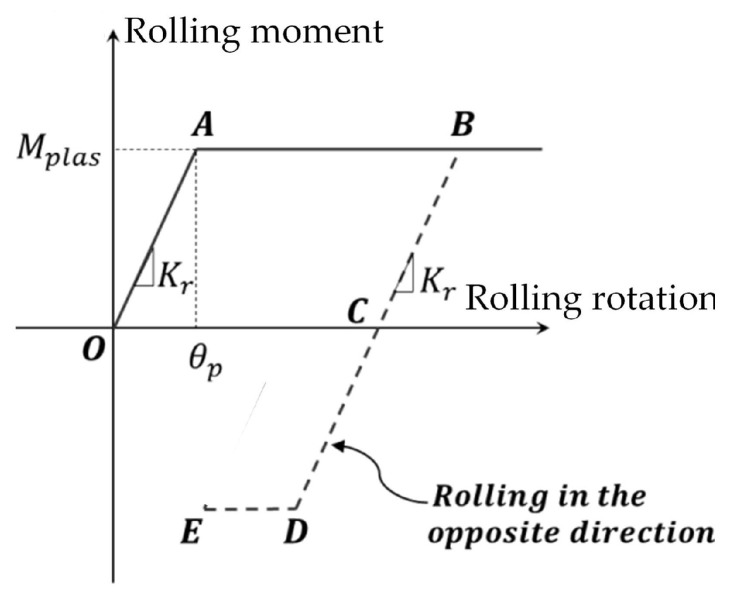
Moment rotation behavior.

**Figure 11:**
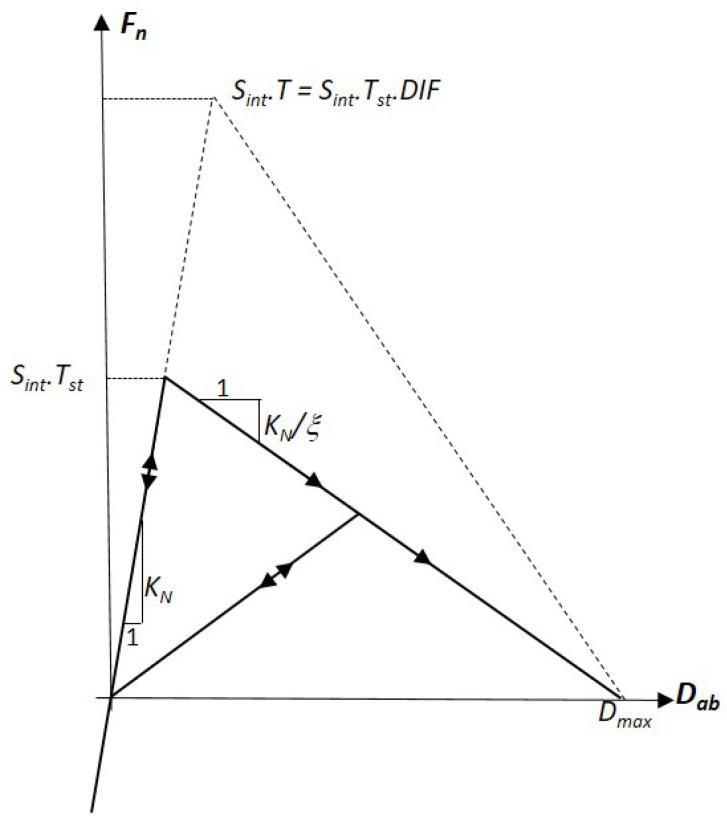
Tensile constitutive behavior.

A rolling resistance is introduced to restrict local rotations and reduce brittle responses [45] (Figure 10). Only the bending resistance is introduced (no torsion). The cohesive link is modeled as a cylindrical beam with radius *r* = Min($R_{a}$, $R_{b}$), and its bending stiffness $K_{r}$ and plastic moment limit $M_{plas}$ are given by:

(5) $$K_{r}=\beta_{r}\frac{E.I}{D_{init}}$$

(6) $$M_{plas}=\eta \frac{T.I}{r}$$

where *T* is the local tensile strength (Figure 11), *I* = $\frac{\pi .r^{4}}{4}$ is the bending inertia. $\beta_{r}$ and η are model parameters controlling ductility.

In a previous study, Potapov et al. [16] demonstrated that the elastic properties of the generated DE assembly remain minimally affected by discretization if the packing technique parameters are kept constant (ratio of maximum to minimum DE radii, ratio of the average DE radius to the average FE size, and the random distribution of DE sizes), thus the dimensionless parameters α,β and γ are already identified. $\beta_{r}$ has almost no influence on the elastic behavior [45].

### 3.4. Failure Criterion

The normal force $F_{n}$ versus displacement $D_{ab}$ diagram in Figure 11 illustrates the non-linear tensile constitutive behavior of the normal interaction between two DE, *a* and *b*. This behavior is considered brittle with no inelastic deformation. Under quasi-static loading, softening begins when the normal force $F_{n}$ reaches $S_{int}T_{st}$, where $T_{st}$ is the static strength. The extent of softening is governed by the coefficient ξ.

The dashed lines represent the tensile constitutive behavior under dynamical loading. In this case, softening initiates when the normal force reaches $S_{int}T$, where *T* is the rate-dependent dynamic strength defined as $T=DIF.T_{st}$. The DIF evolution is presented and identified in Section 4 of the article.

To account for hard-impact scenarios, the DEM model also includes a non-linear compressive constitutive behavior, particularly for modeling the closure porosity under high mean stress [13]. However, this compressive behavior is not detailed in the present study, as the simulations discussed in Section 5 do not involve high mean stress levels.

**Figure 12:**
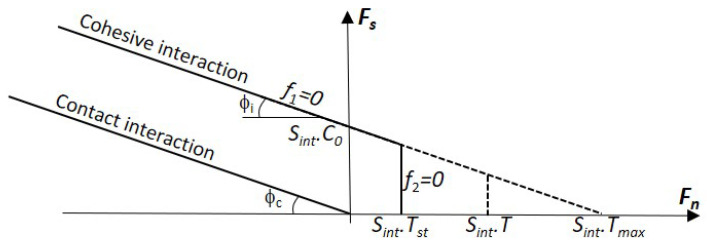
Failure criterion.

Figure 12 presents the modified Mohr–Coulomb failure criterion in the tangential ($F_{s}$) versus normal ($F_{n}$) force plane. The constitutive parameters governing the cohesive interaction are the friction angle $\Phi_{i}$, the cohesion stress $C_{0}$, and the tensile strength *T* ($T=T_{st}$ in case of quasi-static loading). The yield function $f_{1}$ defines the limit for the tangential force, thereby controlling the maximum shear force, while the function $f_{2}$ governs damage under tension, leading up to complete failure of the link when $D_{ab}=D_{max}$. It is important to note that the adopted modified Mohr–Coulomb criterion (Figure 12) imposes an upper limit on the tensile strength, defined as $T_{max}$, which corresponds to the DIF maximum value, $DIF_{max}$ (7).

(7) $$DIF_{max}=\frac{C_{0}}{T_{st}.\tan\phi_{i}}$$

Upon failure of the cohesive interaction, the two involved DE may re-interact if they come into contact. In such a case, a contact interaction is established, characterized by a contact friction angle $\Phi_{c}$. This contact interaction cannot transmit any tensile force.

## 4. Identification of the DEM Model Constitutive Behavior

### 4.1. Identification of Model Parameters for the R30A7 Concrete Under Quasi-Static Loading

The elastic behavior parameters α, β and γ were adopted from Potapov et al. [16], with α=3.9,β=3.75,γ=5.

The non-linear constitutive behavior parameters were identified through simulations of quasi-static uniaxial tensile and compressive tests conducted on R30A7 concrete specimens (Table 1). The influence of each parameter on the macroscopic behavior in both tension and compression was investigated by Omar [45].

**Figure 13:**
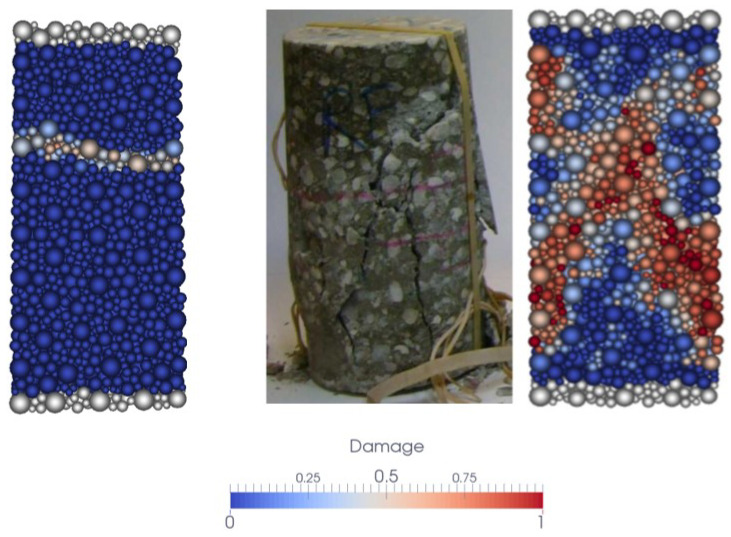
Damage in the sample at the end of uniaxial tests: tension (**left**) and compression (**right**).

Figure 13 shows the final damage distribution in the samples subjected to uniaxial quasi-static tensile and compressive loading. In these simulations, axial displacement was applied at the sample ends over a thin layer of discrete elements. The damage indicator for a DE is defined as the ratio of the number of broken links to the total number of its initial links. In compression, the experimental damage pattern (diagonal cracks) shown in the center of the figure is similar to the one obtained numerically.

**Figure 14:**
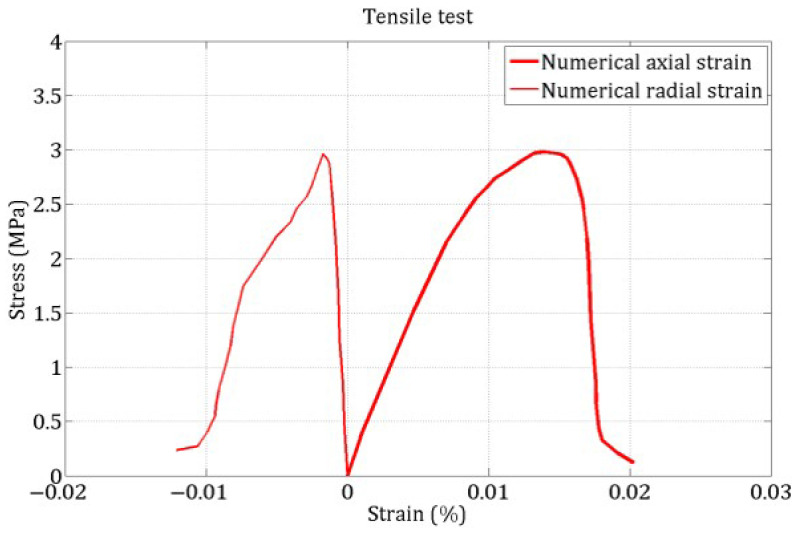
Numerical stress–strain curve in tension.

**Figure 15:**
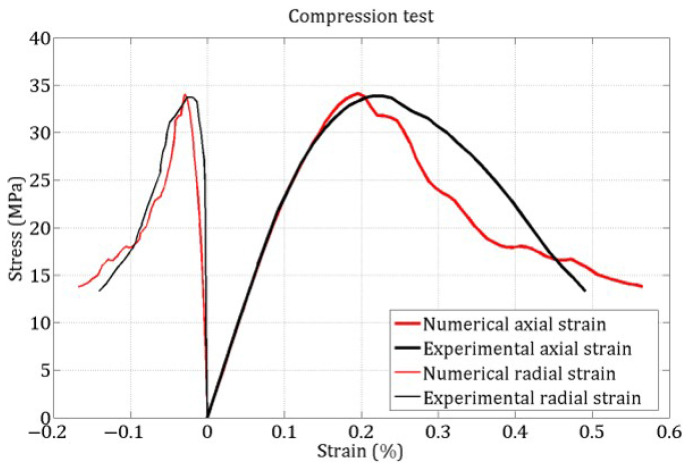
Experimental and numerical stress–strain curve in compression.

Figure 14 and Figure 15 present the stress–strain responses under uniaxial tensile and compressive loading, respectively. Note that no experimental tensile stress–strain curve is available, as the tensile strength was estimated using the Brazilian test.

The accurate reproduction of the tensile peak stress and the compressive stress–strain curve shape enables the identification of the DEM model parameters: $T_{st}=2.1$ MPa, ξ=4, $C_{0}=4$ MPa, $\Phi_{i}=20^{\circ }$, η=5, $\beta_{r}=5$. These tests do not permit the identification of the contact friction angle $\Phi_{c}$, which is therefore assumed equal to $\Phi_{i}$.

### 4.2. Identification of Model Parameters for the R30A7 Concrete Under Tensile Dynamic Loading

After identifying the constitutive behavior parameters under quasi-static loading, this section aims at presenting the DIF evolution. In a first step, the numerical setup and the method used to compute the strain rate are described. Then, the influence of tensile strength is investigated, followed by the proposal of a DIF evolution for normal strength concrete.

#### 4.2.1. Numerical Setup

**Figure 16:**
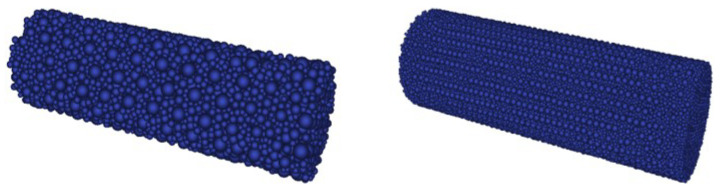
Coarse (**left**) and refined (**right**) DE samples for spalling tests modelling.

To simulate the four spalling tests, two DE concrete samples with different levels of discretization were created, as shown in Figure 16. The characteristics of both assemblies are summarized in Table 3. Isotropy was verified for each assembly. These two DE configurations allow an assessment of the influence of discretization on macroscopic behaviors such as rear face velocity, number of fractures, and their positions. It is worth noting that computational time is approximately proportional to the number of DE.

**Figure 17:**
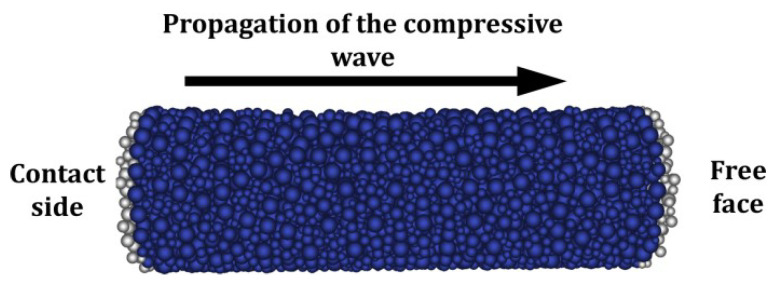
Thin layers of DE used to apply the stress pulse on the contact face and to measure the free face velocity.

**Figure 18:**
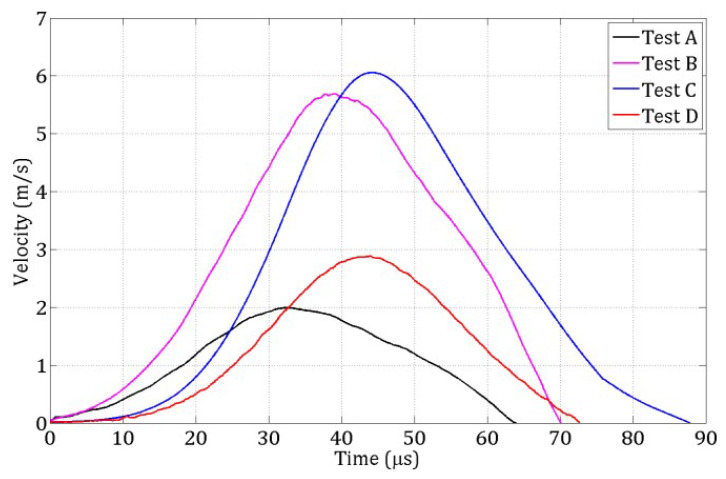
Rear face velocity for the four spalling tests calculated with the coarse discretization.

For each test A, B, C, D (Table 2), the loading pulse (Figure 6) was applied to a 3 mm-thick layer of DE at the contact face, as illustrated in Figure 17. The rear face velocity was computed as the average velocity of DE in a 3 mm-thick layer at the free end. Figure 18 shows this velocity for the coarse discretization.

#### 4.2.2. Calculation of the Strain Rate

The strain rate $\dot{\epsilon }$ in the normal direction $\vec{n}$, between two DE *a* and *b* with velocities $\vec{V_{a}}$ and $\vec{V_{b}}$, and positions $\vec{X_{a}}$ and $\vec{X_{b}}$ is calculated using (8).

(8) $$\dot{\epsilon }=\frac{\vec{V_{b}}-\vec{V_{a}}}{\left|\vec{X_{b}}-\vec{X_{a}}\right|}.\vec{n}$$

To compare the computed strain rate with experimental values, the maximum strain rate during the simulation was extracted. Table 4 presents the maximum values obtained with the coarse discretization. The computed strain rates are significantly higher than those measured experimentally.

**Figure 19:**
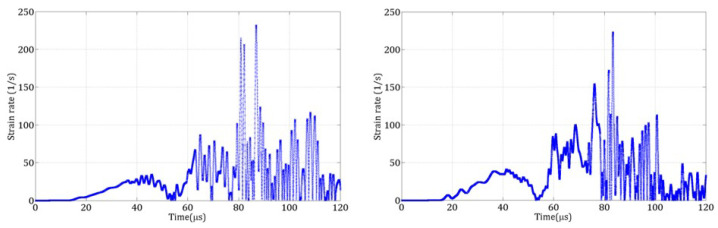
Strain rate evolution in test B for a link located close to the center of the speciment: coarse discretization (**left**), refined discretization (**right**).

To understand these discrepancies, a cohesive link near the center of the specimen was selected for analysis. Figure 19 shows the strain rate evolution for the coarse and refined discretizations. Large oscillations were observed, attributed to the numerical application of the dynamic loading and the use of an explicit time integration scheme. These fluctuations are not physical and must be filtered out.

#### 4.2.3. Strain Rate Filtering

To reduce non-physical oscillations, a temporal filtering technique was implemented, inspired by methods used in explicit solvers such as Altair Radioss [46,47]. The filtered strain rate is computed using (9).

(9) $${\dot{\epsilon }}_{f,n}=\left(1-\alpha \right){\dot{\epsilon }}_{f,n-1}+\alpha {\dot{\epsilon }}_{n}\;$$

where ${\dot{\epsilon }}_{f,n}$ is the filtered strain rate at time $t_{n}$, ${\dot{\epsilon }}_{f,n-1}$ is the filtered strain at time $t_{n-1}$, ${\dot{\epsilon }}_{n}$ is the unfiltered strain rate at time $t_{n}$, $\alpha =2\pi dtF_{f}$ is a smoothing factor between 0 and 1, $F_{f}$ is the filtering frequency and dt is the time step.

Table 5 presents the influence of the filtering frequency $F_{f}$ on the maximum strain rate for test A and for the two discretizations. Based on these results, a filtering frequency of 20 kHz was selected.

#### 4.2.4. Simulations Without Strain-Rate-Dependent Tensile Strength

**Figure 20:**
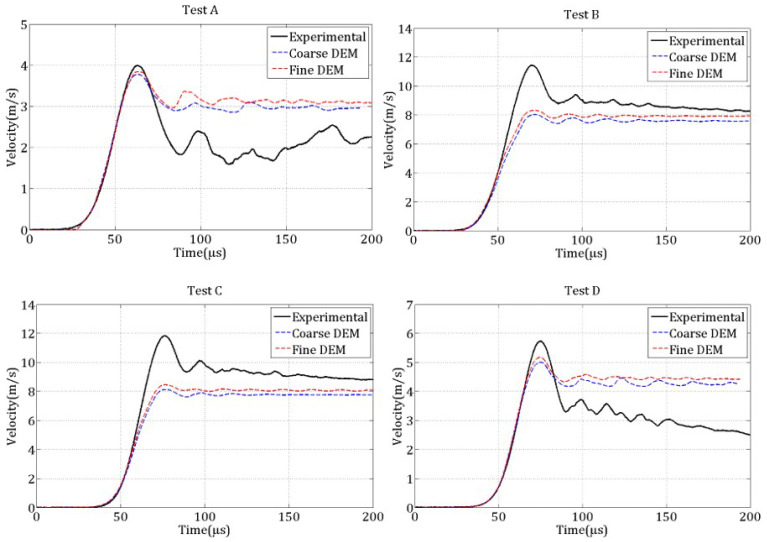
Numerical rear face velocity curves, obtained with no strain rate dependency of tensile strength, compared to the experimental curves for the four spalling tests.

The four spalling tests were initially simulated under the assumption that tensile strength is independent of strain rate (DIF=1). Figure 20 compares the experimental and numerical rear face velocity profiles for each test and both discretizations. The results show poor agreement, especially at higher strain rates, where the numerical curves significantly deviate from the experimental ones. In particular, the pullback velocity $\Delta V_{pb}$ is underestimated, implying an underestimation of the tensile strength according to (1). Furthermore, the peak rear face velocity is too low, potentially due to premature damage induced by radial extensions before the compressive wave reaches the free surface.

These observations suggest that the imposed tensile strength is insufficient, leading to early failure of cohesive links. This indicates the necessity of increasing the local tensile strength as a function of the local strain rate to better capture the dynamic behavior of concrete.

#### 4.2.5. Strain Rate Dependency in Tension

The strain rate sensitivity of concrete in tension arises from the inherent heterogeneity of its microstructure [21,48]. As a result, modeling concrete behavior at the macroscale requires incorporating the influence of strain rate in tension.

Malvar et al. [19] compiled results of tensile tests performed at strain rates up to $10^{2}\;s^{-1}$. When plotted on a log–log scale, the data exhibit a bilinear trend. The first regime corresponds to a moderate increase in strength with strain rate, while the second regime shows a more pronounced increase. The transition between these two regimes occurs around $10^{0}\;s^{-1}$. Furthermore, the slopes of both regimes depend on the compressive strength of the concrete.

Inspired by the CEB formulation [49] and Malvar et al. [19], the DIF evolution of the DEM model is given in (10) and (11). ${\dot{\epsilon }}_{st}$ and ${\dot{\epsilon }}_{m}$ are the quasi-static and moderate strain rates, $\sigma_{c}$ represents the concrete compressive strength, and $\sigma_{c0}$ (set at 10 MPa) serves as a reference value. Parameters $\delta_{1}$ and $\delta_{2}$ are the slopes of the first and second regimes.

(10) $$DIF=\frac{T}{T_{st}}=\left\{\begin{array}{cc} 1 & \mathrm{if}\;\dot{\epsilon }\leq {\dot{\epsilon }}_{st}\\ {\left(\frac{\dot{\epsilon }}{{\dot{\epsilon }}_{st}}\right)}^{\delta_{1}} & \mathrm{if}\;{\dot{\epsilon }}_{st}\leq \dot{\epsilon }\leq {\dot{\epsilon }}_{m}\\ \theta {\left(\frac{\dot{\epsilon }}{{\dot{\epsilon }}_{st}}\right)}^{\delta_{2}} & \mathrm{if}\;{\dot{\epsilon }}_{m}\leq \dot{\epsilon } \end{array}\right.$$

(11) $$\mathrm{where}\;\delta_{1}=\frac{1}{1+8\frac{\sigma_{c}}{\sigma_{0}}}\;\;\mathrm{and}\;\;\ln\theta =6\delta_{1}-2$$

Equations (10) and (11) allow calculation of the DIF for any given strain rate $\dot{\epsilon }$. Based on the parameters identified in previous sections and the upper limit imposed by (7), the maximum value of the DIF is approximatively 5, which is consistent with experimental findings reported in the literature [19].

## 5. Discussion on the Simulations of the Four Spalling Tests with the Updated DEM

**Figure 21:**
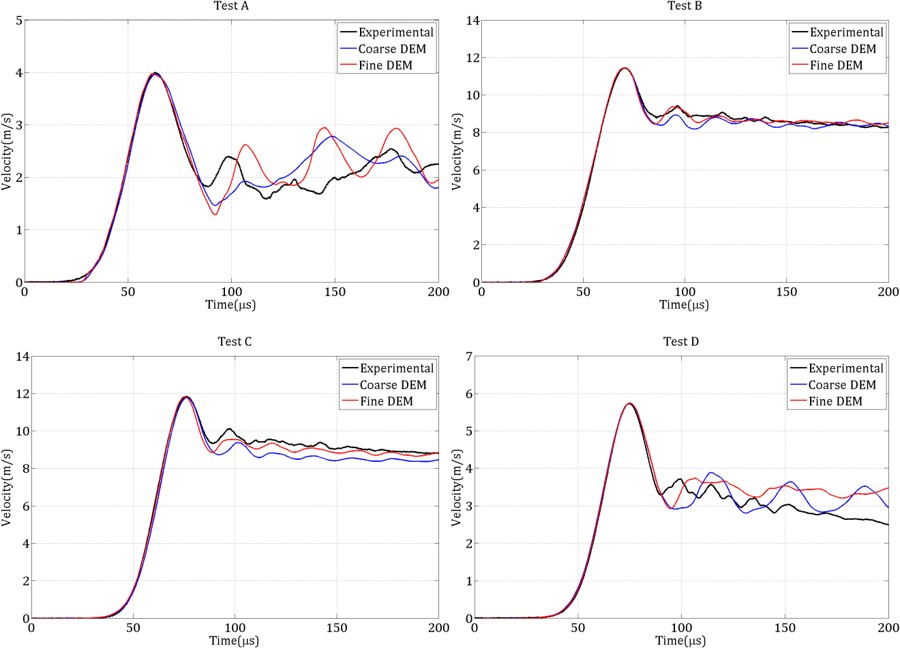
Numerical rear-face velocity curves, obtained with local strain rate dependency of tensile strength, compared to the experimental curves for the four spalling tests.

The four spalling tests by Erzar [24,25] were simulated taking into account the temporal filtering technique (9), the constitutive behavior parameters identified under quasi-static loading and the strain rate dependency of the tensile strength (10) and (11). Figure 21 presents the rear face velocity curves for the four tests and both discretizations. The numerical results show good agreement with the experimental data, particularly regarding the peak velocity and the first rebound. The coarse discretization also yields satisfactory results, demonstrating the robustness of the DEM model in reproducing the macroscopic experimental behavior across different levels of refinement.

In test A, the peak velocity is well captured, while the rebound velocity (approximately 1.5 m/s) is slightly underestimated compared to the experimental value of 1.85 m/s. This corresponds to a relative error in tensile strength of about 16%. The post-rebound velocity profile exhibits oscillations, indicating that a portion of the elastic waves is trapped between the damaged zone and the rear face of the specimen. For tests B, C, and D, the relative error on the pullback velocity—and hence on the inferred tensile strength—is smaller. The velocity signals contain fewer oscillations, suggesting that most of the elastic energy was dissipated during the fracturing process.

**Figure 22:**
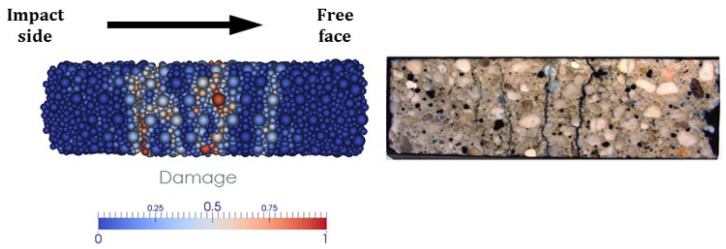
Test B: comparison of numerical damage at 1.3 ms and experimental observation after the test [24].

As previously mentioned, the position of the fracture plane is not a reliable indicator of dynamic tensile strength and, therefore, is not suitable for model validation. However, the numerical damage field can be compared with experimental observations. Figure 22 shows the damage field in the specimen with the coarse discretization for test B at time t=1.3 ms, which is well after the failure time. The simulation reveals the formation and propagation of microcracks that eventually coalesce into macrocracks, especially near the specimen’s midsection. These macrocracks merge to form rupture planes that divide the sample into distinct fragments. This is consistent with experimental observations, where a primary fracture plane is visible alongside several secondary macrocracks.

**Figure 23:**
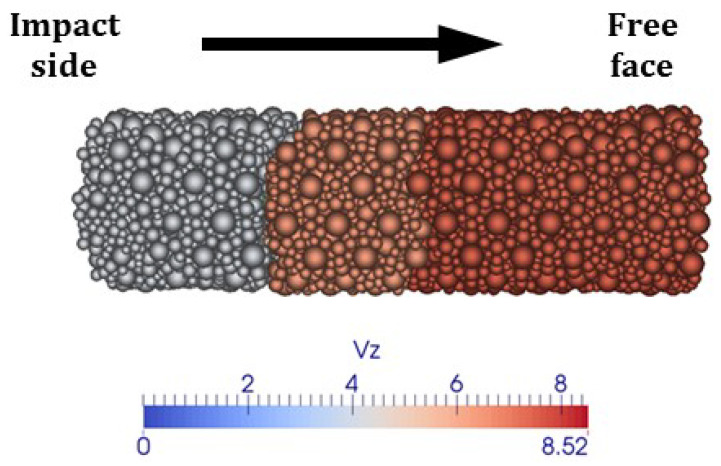
Test B: axial velocity at 1.3 ms.

Additionally, the axial velocity field at t=1.3 ms, shown in Figure 23, clearly indicates the separation of the sample into distinct pieces.

## 6. Conclusions

This study presents an enhanced 3D DEM model tailored to capture the dynamic tensile behavior of concrete at high strain rates. The key innovation lies in the introduction and validation of a strain-rate-dependent tensile strength within the microscale cohesive interactions between discrete elements.

While the strain-rate effects observed in dynamic compression can be largely attributed to inertial phenomena, this work demonstrates that accurately modeling tensile damage under dynamic loading requires embedding strain-rate sensitivity at the microscale. To this end, the DEM model was first calibrated under quasi-static loading and then extended to include a dynamic increase factor formulation inspired by existing empirical models.

The model was validated against four spalling tests on normal-strength concrete specimens, covering a range of strain rates typical of hard impact scenarios (30–115 $s^{-1}$). The DEM simulations, using both coarse and refined discretizations, successfully reproduced key experimental indicators such as rear-face velocity profiles and fracture patterns.

The present results support the relevance of the proposed modeling approach for simulating tensile damage in concrete under high-rate dynamic loading. The study contributes to ongoing efforts to improve the predictive capability of the DEM model for assessing the performance of concrete structures subjected to severe dynamic events.

## Figures and Tables

**Table 1:** Composition and mechanical properties of wet R30A7 concrete [41].

Composition		Mechanical Properties	
Cement CEM I 52.5N	263 ${\mathrm{kg.m}}^{-3}$	Porosity	12%
Sand 0–1.8 mm	838 ${\mathrm{kg.m}}^{-3}$	Slump	7 cm
Gravel 0.5–8 mm	1008 ${\mathrm{kg.m}}^{-3}$	Young modulus *E*	25 GPa
Water	169 ${\mathrm{kg.m}}^{-3}$	Poisson ratio ν	0.16
W/C ratio	0.64	Compressive strength $\sigma_{c}$	34 MPa
Density	2280 ${\mathrm{kg.m}}^{-3}$	Tensile strength $\sigma_{t}$	3.0 MPa
		Saturation degree	42%

**Table 2:** Strain rates at failure for the four spalling tests carried out on wet R30A7 concrete [24,25].

Test	A	B	C	D
$\dot{\epsilon }$ ($s^{-1}$)	30	105	115	50

**Table 3:** Characteristics of the two DE assemblies.

Characteristics	Coarse Discretization	Refined Discretization
Number of DE	6744	60,323
Mean radius (mm)	1.5	0.7
Compactness	0.58	0.62

**Table 4:** Numerical and experimental maximum values of strain rates for the four spalling tests.

Test	A	B	C	D
Experimental ${\dot{\epsilon }}_{max}$ ($s^{-1}$)	30	105	115	50
Numerical ${\dot{\epsilon }}_{max}$ ($s^{-1}$)	120	260	280	140

**Table 5:** Influence of filtering frequency on the maximum strain rate for test A (${\dot{\epsilon }}_{max}$ = 30 $s^{-1}$).

$F_{f}$ (kHz)	Coarse Discretization ${\dot{\epsilon }}_{\max}$ ($s^{-1}$)	Refined Discretization ${\dot{\epsilon }}_{\max}$ ($s^{-1}$)
10	24	28
20	29	33
50	34	43

## Data Availability

The original contributions presented in this study are included in the article. Further inquiries can be directed to the corresponding author.

## Abbreviations

The following abbreviations are used in this manuscript:

1D	One-dimensional
3D	Three-dimensional
DE	Discrete element
DEM	Discrete element method
DIF	Dynamic increase factor
FE	Finite element
FEM	Finite element method
SHPB	split-Hopkinson pressure bar
